# Bispecific antibody therapy in CNS myeloma: Early evidence from a multicentre cohort

**DOI:** 10.1111/bjh.70108

**Published:** 2025-08-26

**Authors:** Alexandra Noveihed, Samer Al Hadidi, Meera Mohan, Mansi R. Shah

**Affiliations:** ^1^ Rutgers Robert Wood Johnson Medical School New Brunswick New Jersey USA; ^2^ Myeloma Center University of Arkansas for Medical Sciences Little Rock Arkansas USA; ^3^ Medical College of Wisconsin Milwaukee Wisconsin USA; ^4^ Rutgers Cancer Institute New Brunswick New Jersey USA

**Keywords:** bispecific antibodies, CNS myeloma, multiple myeloma, myelomatous meningitis, talquetamab, teclistamab

To the Editor,

Extramedullary disease (EMD) occurs in 5%–10% of all multiple myeloma (MM) cases, with central nervous system (CNS) involvement comprising approximately one‐fifth of EMD presentations.^1^, ^2^ CNS‐MM may occur at relapse or, less commonly, de novo at diagnosis. Both forms are associated with poor outcomes. Historically, median overall survival (OS) after CNS involvement was 4–7 months, although this OS has improved to approximately 12 months after 2016 with the advent of myeloma therapies.^3^, ^4^, ^5^ The rising incidence of CNS‐MM may reflect improved imaging, longer survival with novel therapies and clonal evolution leading to atypical relapse patterns.^6^ Patients with CNS‐MM are typically younger and more likely to harbour high‐risk cytogenetic abnormalities.^1^, ^3^, ^7^

CNS‐MM represents a rare but aggressive disease manifestation with limited evidence‐based treatment guidelines. Systematic investigation of therapeutic interventions has been hampered by the routine exclusion of these patients from prospective clinical trials, necessitating reliance on retrospective analyses for clinical decision‐making.^1^ Additionally, the blood–brain barrier presents a significant pharmacological challenge, as most conventional anti‐myeloma agents demonstrate poor CNS penetration, resulting in subtherapeutic cerebrospinal fluid (CSF) concentrations. Current management strategies employ multimodal therapeutic approaches integrating focal or whole‐brain radiation therapy (RT), intrathecal (IT) chemotherapy administration and systemic treatments.^1^, ^4^, ^8^

Despite demonstrating modest blood–brain barrier permeability in pharmacokinetic studies, immunomodulatory drugs such as lenalidomide and pomalidomide, as well as the anti‐CD38 monoclonal antibody daratumumab, frequently exhibit limited efficacy in the CNS‐MM setting. This therapeutic resistance is often attributable to prior drug exposure in heavily pretreated patients, leading to the development of refractory disease characterized by complex clonal evolution and altered biological characteristics of the malignant plasma cells.^9^, ^10^

Bispecific antibodies (BsAbs) have demonstrated high efficacy in relapsed/refractory MM (RRMM), with overall response rates ranging from 60% to 70%, median progression‐free survival of 11.4–21 months and OS ranging from 1 to 2 years. However, their role in CNS‐MM remains inadequately characterized. Currently, two BsAb classes have received United States Food and Drug Administration (FDA) approval for RRMM: targeting B‐cell maturation antigen (BCMA) and those targeting G‐protein–coupled receptor family C group 5 member D (GPRC5D). Notably, the pivotal clinical trials that led to these approvals systematically excluded patients with active CNS involvement.^1^ The emerging clinical evidence supporting BsAb efficacy in CNS‐MM is limited to isolated case reports, including successful treatment with elranatamab^11^ and comparative data from BCMA‐targeted chimeric antigen receptor T‐cell (CAR‐T) therapy, which demonstrated a 100% CNS response rate in a retrospective analysis.^12^ Despite this promising preliminary evidence for CAR‐T approaches in CNS‐MM, the extended manufacturing timeframe required for these cellular therapies may substantially limit their clinical application in patients presenting with rapidly progressive CNS disease.

We conducted a multicentre retrospective analysis of patients with CNS‐MM treated with BsAbs at three academic medical centres in the United States. CNS‐MM was defined by the presence of plasmacytomas (brain parenchymal or optic nerve lesions), leptomeningeal enhancement on imaging or plasma cell meningitis confirmed by CSF cytology.

Nine patients with CNS‐MM treated with either BCMA or GPRC5D BsAbs were included. Eight patients received talquetamab (GPRC5D‐directed); one received teclistamab (BCMA‐directed).

Individual characteristics of the cohort are included in Table 1. The median age at CNS‐MM diagnosis was 66 years (range 48–73 years); 66.7% were male. High‐risk cytogenetics [*t(4;14)*, *t(14;16)*, *t(14;20)*, *gain/amp(1q21)*, *del(1p)* and/or *del(17p)*] were present in 77.8% of patients. Additional EMD was present in four patients (44.4%), and plasma cell leukaemia was identified in 22.2% (*n* = 2). Most patients were penta‐refractory (77.8%), defined as resistance to five major anti‐myeloma therapies: lenalidomide, pomalidomide, bortezomib, carfilzomib and either daratumumab or isatuximab. All patients had received prior autologous stem cell transplant (autoHSCT), and 44.4% had prior BCMA‐targeted CAR‐T therapy.

**TABLE 1:** Individual patient characteristics.

Case #	Age at MM dx	Sex	MM isotype	Cytogenetics^a^	Induction therapy	Prior AutoSCT	Prior CAR‐T	Prior lines of therapy before BsAb	Time from MM dx to CNS dx (months)	Tx prior to CNS dx	Type of CNS disease	Type of bispecific	Additional CNS treatment modality utilized?
1	67	F	IgA Kappa	*1q21*	VRd	Yes	Ide‐Cel	6	37	Ide‐cel	Plasmacytoma	Talquetamab	RT
2	73	M	IgA Kappa	*1q21*	D‐VRd	Yes	No	5	13	Carfilzomib/Daratumumab/Dexamethasone	Plasmacytoma	Talquetamab	RT
3	62	M	IgG Lambda	*1q21, del(17p)*	KCyd	Yes	No	4	29	Daratumumab and Revlimid Maintenance	Plasmacytoma and meningitis	Talquetamab	RT and IT triple therapy^b^
4	52	M	IgG Lambda	*1q21, del(17p), del(1p)*	VRD	Yes	Ide‐Cel	7	132	Elotuzumab/Bortezomib/Dexamethasone	Meningitis	Talquetamab	RT and IT with chemotherapy with MTX/Cytarabine alternating
5	67	M	Kappa Light Chain	N/A	VRD	Yes	No	6	64	Teclistamab	Meningitis	Talquetamab	RT and IT chemotherapy with MTX/Cytarabine alternating
6	66	F	IgA Lambda	*t(4;14) 1q21*	D‐KRd	Yes	Cilta‐Cel	6	141	Cilta‐cel	Plasmacytoma and meningitis	Talquetamab	RT and IT triple therapy
7	48	F	IgG Kappa	*1q21*	CyBorD	Yes	Ide‐Cel	8	73	PACMED with Selinexor	Plasmacytoma and meningitis	Talquetamab	RT and IT triple therapy
8	65	M	IgG Kappa	*del(17p)*	VTD‐Pace	Yes	No	9	94	Belantamab‐mafodotin	Plasmacytoma	Talquetamab	RT
9	71	M	IgG Kappa	Hyperdiploidy	VTD‐Pace	Yes	No	7	116	Belantamab‐mafodotin	Plasmacytoma and meningitis	Teclistamab	No

Abbreviations: CAR‐T, chimeric antigen receptor T‐cell; Cilta‐cel, ciltacabtagene autoleucel; CNS, central nervous system; CyBorD, cyclophosphamide, bortezomib and dexamethasone; D‐KRd, daratumumab, carfilzomib, lenalidomide and dexamethasone; D‐VRd, daratumumab, bortezomib, lenalidomide and dexamethasone; Ide‐cel, idecabtagene vicleucel; IT, intrathecal; KCyd, carfilzomib, cyclophosphamide and dexamethasone; MM, multiple myeloma; PACMED, cisplatin, cytarabine, cyclophosphamide, mesna, etoposide and dexamethasone; RT, radiation therapy; VRd, bortezomib, lenalidomide and dexamethasone; VTD‐PACE, bortezomib, thalidomide, dexamethasone, cisplatin, doxorubicin, cyclophosphamide and etoposide.

^a^ Cytogenetic abnormalities evaluated: FISH for *t(11;14)*, *t(4;14)*, *t(14;16)*, *t(14;20)*, *del(17p)*, *1q21*, *del(1p)*, hyperdiploidy. Presence of *t(4;14)*, *t(14;16)*, *t(14;20)*, *gain/amp(1q21)*, *del(1p)* and/or *del(17p)* indicates high risk.

^b^ IT triple therapy: a combination of methotrexate (MTX), cytarabine and hydrocortisone injection.

CNS response, defined as stability or reduction in the size of intracranial or optic plasmacytoma on radiographic evaluation or reduction in the number of plasma cells in the CSF, was evaluated. Three patients were excluded from response assessment due to early mortality, transition to hospice care or insufficient follow‐up. Among the six evaluable patients, the CNS overall response rate was 100%. Key aspects of the clinical courses in the evaluable patients (*n* = 6) are included in Table 2, with the CNS non‐responder clinical courses summarized in Table 3. Median time from MM diagnosis to CNS relapse was 65.5 months (range: 15–131 months) among the CNS responders versus 73 months (range: 64–141 months) among the non‐responders. Median time to best CNS response was 10 weeks (range: 1–20 weeks), occurring after a median of 6.5 BsAb doses (range: 4–10 doses). Notably, two patients demonstrated CNS responses prior to the initiation of adjunctive CNS‐directed therapies. One patient (Table 1: Case 4) was diagnosed with myelomatous meningitis after CSF analysis demonstrated 498 clonal plasma cells on cytological examination. Following initiation of talquetamab with step‐up dosing, the CSF plasma cell count decreased to 74 cells within 1 week, prior to the implementation of IT chemotherapy or RT. This patient subsequently received combined IT chemotherapy and continued talquetamab therapy, resulting in complete resolution of CNS involvement. An additional patient (Table 1: Case 9) received Teclistamab for CNS plasmacytoma and myelomatous meningitis and demonstrated CNS response radiographically after 12 weeks without the addition of CNS‐directed therapies.

**TABLE 2:** Clinical courses for CNS responders.

Case #	Type of CNSdisease	Type of bispecific	Best systemic response^a^	CRS grade	ICANS grade	Time to best CNS response (weeks)^b^	Additional CNS treatment modality utilized?	Time on bispecific by data cut‐off date (months)^d^	Relapse or progression after BsAb?^d^
1	Plasmacytoma	Talquetamab	VGPR	2	0	20	RT	11	No
2	Plasmacytoma	Talquetamab	CR	1	0	8	RT	4	No
3	Plasmacytoma and meningitis	Talquetamab	CR	0	0	1	RT and IT triple therapy^c^	6	Yes
4	Meningitis	Talquetamab	Not evaluable	0	0	5	RT and IT with chemotherapy with MTX/Cytarabine alternating	2	No
8	Plasmacytoma	Talquetamab	CR	0	0	12	RT	12	No
9	Plasmacytoma and meningitis	Teclistamab	CR	0	0	12	No	6	No

Abbreviations: BsAb, bispecific antibodies; CNS, central nervous system; CR, complete response based on IMWG Response Criteria; CRS, cytokine release syndrome; ICANS, immune effector cell–associated neurotoxicity syndrome; IT, intrathecal; RT, radiation therapy; VGPR, very good partial response based on IMWG Response Criteria.

^a^ Best systemic response: based on International Myeloma Working Group (IMWG) Uniform Response Criteria of 2016. No other systemic therapies (IMiDs, chemotherapy, proteasome inhibitors, anti‐CD38 monoclonal antibodies) were administered.

^b^ Time to best CNS response: defined as number of weeks from first BsAb administration to CNS response.

^c^ IT triple therapy: a combination of methotrexate (MTX), cytarabine and hydrocortisone injection.

^d^ Data cut‐off date is 31 December 2024 apart from Patient 6 who was added with data cut‐off date of 31 March 2025. Additional follow‐up data are unavailable for our review.

**TABLE 3:** Clinical courses for CNS non‐responders.

Case #	Type of CNS disease	Type of bispecific	Best systemic response^a^	CRS grade	ICANS grade	Time to best CNS response (weeks)^b^	Additional CNS treatment modality utilized?	Time on bispecific by data cut‐off date (months)^d^	Relapse or progression after BsAb?^d^
5	Meningitis	Talquetamab	Not evaluable	0	0	N/A	RT and IT chemotherapy with MTX/Cytarabine alternating	3	Deceased
6	Plasmacytoma and meningitis	Talquetamab	Not evaluable	0	0	N/A	RT and IT triple therapy^c^	1	No
7	Plasmacytoma and meningitis	Talquetamab	CR	1	0	N/A	RT and IT triple therapy	6	Deceased

Abbreviations: BsAb, bispecific antibodies; CNS, central nervous system; CR, complete response per IMWG Uniform Response Criteria; CRS, cytokine release syndrome; ICANS, immune effector cell–associated neurotoxicity syndrome; IT, intrathecal; RT, radiation therapy.

^a^ Best systemic response: based on International Myeloma Working Group (IMWG) Uniform Response Criteria of 2016. No other systemic therapies (IMiDs, chemotherapy, proteasome inhibitors, anti‐CD38 monoclonal antibodies) were administered.

^b^ Time to best CNS response: defined as number of weeks from first BsAb administration to CNS response.

^c^ IT triple therapy: a combination of methotrexate (MTX), cytarabine and hydrocortisone injection.

^d^ Data cut‐off date is 31 December 2024 apart from Patient 6 who was added with data cut‐off of 31 March 2025. Additional follow‐up data are unavailable for our review.

Systemic responses were evaluated based on the International Myeloma Working Group (IMWG) Uniform Response Criteria. There were six patients with evaluable systemic responses after BsAb (Table 2 and Table 3) with 100% attaining very good partial response (VGPR) or better. Supplementary CNS‐directed therapy—including radiation and/or IT chemotherapy—was administered to 88.9% of patients in the entire cohort. Cytokine release syndrome (CRS) was reported in 44.4% of patients and was limited to grade 1 (*n* = 3) or grade 2 (*n* = 1) in severity. No patients experienced grade ≥3 CRS or any immune effector cell–associated neurotoxicity syndrome (ICANS).

This is the first retrospective study reporting CNS responses following BsAb therapy in patients with CNS‐MM. Notably, two patients exhibited CNS improvement prior to the initiation of adjunctive therapy, raising the possibility of intrinsic CNS activity with BsAb monotherapy. These findings provide preliminary evidence supporting the potential CNS efficacy of BsAbs in heavily pretreated patients with MM, suggesting that these agents may penetrate the blood–brain barrier and exert therapeutic activity within the CNS compartment. In our series, responses were observed predominantly with GPRC5D‐targeted BsAbs; however, most patients in the cohort received talquetamab.

In terms of safety, no patients developed ICANS, and CRS events were limited to grade 1–2 events. These findings are notable given concerns that CNS involvement could predispose to neurotoxicity with T‐cell–redirecting therapies. In CD19 CAR‐T studies in lymphoma, CNS disease has been associated with increased ICANS risk,^13^ but comparable safety profiles in myeloma‐specific therapies have not been previously reported. The relatively low incidence of neurotoxicity observed with BsAbs may, in part, reflect the limited expression of their target antigens in normal brain tissue. BCMA expression is virtually undetectable levels in normal brain tissue,^14^ suggesting minimal risk for on‐target neurotoxicity with BCMA‐directed therapies. Similarly, GPRC5D mRNA has been detected at low levels in motor neurons of the inferior olivary nucleus by in situ hybridization, but without corresponding protein expression by immunohistochemistry; neither GPRC5D mRNA nor protein has been detected in the cerebral cortex, basal ganglia, midbrain or cerebellum.^15^

CAR‐T therapy has demonstrated efficacy in CNS‐MM, with reported CRS and ICANS rates comparable to patients without CNS disease.^12^ However, its use is constrained by a complex, time‐intensive manufacturing process requiring several weeks from apheresis to infusion—a delay that may be untenable for patients with aggressive CNS progression. In contrast, BsAbs targeting the same tumour antigens are available off‐the‐shelf, permitting immediate treatment initiation. This temporal advantage may be particularly important in CNS‐MM, where rapid disease control can be essential to prevent irreversible neurological complications and mortality. Additionally, BsAb therapy offers dosing flexibility, allowing for modification or discontinuation in response to adverse events—an option not available with single‐infusion CAR‐T products.

In conclusion, BsAbs may represent a viable therapeutic option for heavily pretreated patients with CNS‐MM, demonstrating potential for achieving rapid CNS disease control without increased risk of severe CRS or ICANS. Limitations to our study include difficulty in distinguishing CNS response directly attributed to BsAb as most patients in our cohort were treated with CNS‐directed therapies such as IT chemotherapy or RT. However, two patients were able to achieve CNS responses either prior to the initiation of or despite the omission of CNS‐directed therapies. Furthermore, our study suggests the addition of BsAb to CNS‐directed therapies is a safe and effective treatment approach for CNS‐MM patients. Additional limitations of our study include the small sample size and short follow‐up. Prospective studies with larger cohorts, standardized CNS response criteria and longer observation are warranted to better define the efficacy, safety and durability of BsAb therapy in this setting. Nonetheless, these findings provide preliminary support for the clinical utility of BsAbs in managing CNS manifestations of MM.

## AUTHOR CONTRIBUTIONS

A.N. and M.R.S. designed the study and analysed the data. S.A.H., M.M. and M.R.S. were involved in the treatment of these patients. All authors collected the data, contributed to writing the manuscript, critically reviewed the manuscript and approved its final revision.

## FUNDING INFORMATION

This research has received no external funding.

## CONFLICT OF INTEREST STATEMENT

A.N. declares no conflict of interest. M.S. declares conflicts of interest including: Abbvie: Research Funding; Johnson and Johnson: Research funding, Honoraria, Consultancy, Speaker; Regeneron: Research funding, Travel stipend, Consultancy, Speaker; Bristol‐Myers Squibb: Research funding, Honoraria; Targeted Oncology: Honoraria; SWOT: Honoraria; Pfizer: Honoraria, Consultancy; Aptitude Health: Honoraria. S.A.H. declares: Janssen: Research funding, Consultancy; Pfizer: Research funding; Sanofi: Consultancy. M.M. declares: Sanofi: Research funding, Consultancy; Bristol‐Myers Squibb: Research funding, Consultancy; Jannsen: Consultancy; Pfizer: Consultancy; Legend Biotech: Consultancy; Adaptive Biotech: Consultancy. M.M. also declares Research grants from MCW CTSI and Advancing Healthier Wisconsin KL2 Award as well as the Advancing Healthier Wisconsin Pilot Grant.

## PATIENT CONSENT STATEMENT

Complete informed consent was obtained from patients in this study.

## Supporting information


Data S1.

## Data Availability

For original data, contact Dr. Mansi R Shah.
