# Supplementary material for: Bispecific antibody therapy in CNS myeloma: Early evidence from a multicentre cohort
Source: Br J Haematol. 2025 Aug 26;207(5):2161–6. doi: 10.1111/bjh.70108 (PMC12624187; doi:10.1111/bjh.70108)
Supplement: Supplementary file 1 — Data S1. [file BJH-207-2161-s001.docx]

**Supplementary References For Manuscript Entitled “Bispecific Antibody Therapy in CNS Myeloma: Early Evidence from a Multicenter Cohort”**

Alexandra Noveihed, Samer Al Hadidi, Meera Mohan, Mansi R. Shah

**Additional References**

1. Chari A, Minnema Monique C, Berdeja Jesus G, Oriol A, van de Donk Niels WCJ, Rodríguez-Otero P, et al. Talquetamab, a T-Cell–Redirecting GPRC5D Bispecific Antibody for Multiple Myeloma. New England Journal of Medicine. 2022;387(24):2232-44.
2. Moreau P, Garfall AL, Donk NWCJvd, Nahi H, San-Miguel JF, Oriol A, et al. Teclistamab in Relapsed or Refractory Multiple Myeloma. New England Journal of Medicine. 2022;387(6):495-505.
3. Lesokhin AM, Tomasson MH, Arnulf B, Bahlis NJ, Miles Prince H, Niesvizky R, et al. Elranatamab in relapsed or refractory multiple myeloma: phase 2 MagnetisMM-3 trial results. Nature Medicine. 2023;29(9):2259-67.
4. Chng WJ, Dispenzieri A, Chim CS, Fonseca R, Goldschmidt H, Lentzsch S, et al. IMWG consensus on risk stratification in multiple myeloma. Leukemia. 2014;28(2):269-77.
5. Muscal JA, Sun Y, Nuchtern JG, Dauser RC, McGuffey LH, Gibson BW, et al. Plasma and cerebrospinal fluid pharmacokinetics of thalidomide and lenalidomide in nonhuman primates. Cancer Chemother Pharmacol. 2012;69(4):943-7.
6. Rasche L, Bernard C, Topp MS, Kapp M, Duell J, Wesemeier C, et al. Features of extramedullary myeloma relapse: high proliferation, minimal marrow involvement, adverse cytogenetics: a retrospective single-center study of 24 cases. Ann Hematol. 2012;91(7):1031-7.
7. Gust J, Hay KA, Hanafi LA, Li D, Myerson D, Gonzalez-Cuyar LF, et al. Endothelial Activation and Blood-Brain Barrier Disruption in Neurotoxicity after Adoptive Immunotherapy with CD19 CAR-T Cells. Cancer Discov. 2017;7(12):1404-19.
8. Zweegman S, van de Donk NWCJ. Deletion 17p: a matter of size and number? Blood. 2021;137(9):1135-6.
